# A systematic review of CD14 and toll-like receptors in relation to asthma in Caucasian children

**DOI:** 10.1186/1710-1492-9-10

**Published:** 2013-03-15

**Authors:** Ester MM Klaassen, Brenda EJT Thönissen, Guillaume van Eys, Edward Dompeling, Quirijn Jöbsis

**Affiliations:** 1 Department of Paediatric Pulmonology, School for Public Health and Primary Care (CAPHRI), Maastricht University Medical Centre (MUMC), P.O. Box 5800, Maastricht, 6202 AZ, the Netherlands; 2 Department of Genetics and Cell Biology, Cardiovascular Research Institute Maastricht (CARIM), MUMC, P.O. Box 5800, Maastricht, 6202 AZ, the Netherlands

**Keywords:** Asthma, Caucasian, CD14, Children, Gene expression, Genetic variants, Polymorphisms, TLR, Wheeze

## Abstract

The aetiology of childhood asthma is complex. An early dysfunction in the immunological development of the innate immune system in combination with environmental factors possibly triggers asthma. CD14 and toll-like receptors are important components of the innate immune system. The aim of this systematic review was to obtain a better insight into the relation between CD14 and toll-like receptors and childhood asthma in Caucasians. We searched PubMed and EMBASE for relevant articles. In total, 44 articles were included. The quality of the selected studies was independently assessed by the first two authors using the Newcastle-Ottawa quality assessment scale. Toll-like receptor 2, toll-like receptor 6, toll-like receptor 9, and toll-like receptor 10 appear to have some association with childhood asthma in Caucasians. The evidence for a relation of CD14 with childhood asthma is limited. In conclusion, there is no convincing evidence yet for a role of CD14 and toll-like receptors in relation to childhood asthma. Future studies should include haplotype analysis and take environmental factors into account to further clarify the role of CD14 and toll-like receptors on childhood asthma.

## Review

### Background

Asthma is a common disease in childhood and is characterized by chronic eosinophilic airway inflammation and airway (hyper) responsiveness [1]. Both, genetic and environmental factors have been associated with asthma. The cumulative effect of these factors is large, though the individual contribution of each factor may be limited [2-6]. Also, genetic and environmental factors can modulate each other. As this modulation can differ with age, it can be time dependent [3]. Hence, the aetiology of childhood asthma is complex.

It is expected that an early dysfunction in the immunological development of the innate immune system, in combination with environmental factors may trigger childhood asthma [7]. The innate immune system makes the first contact with pathogens. Genetic variations in components of the innate immune system can alter the capability to deal with pathogens. The direction of the innate immune system response can depend on presented environmental signals, like endotoxin exposure [8]. The difference in genetic make-up combined with differences in environmental factors leads to variations in the immune response. Important components of the innate immune system are CD14, an adaptor molecule, and a system of pathogen receptors named toll-like receptors (TLRs) [9].

CD14 is a multifunctional high-affinity receptor for endotoxins, lipopolysaccharides and other bacterial wall components. It has been implicated in the development and maturation of the innate immune system [10-13]. Several studies have associated CD14 with determination of the balance between Th1 versus Th2 cytokines [14-16]. It is expressed on the surface of monocytes, macrophages, and neutrophils and occurs as a membrane-bound form and a soluble form [17,18]. Polymorphisms in *CD14* and levels of soluble CD14 (sCD14) have been implicated in childhood asthma [10,19-23].

TLRs are evolutionarily conserved receptor complexes in the pathogen-recognition process [24]. Each of the ten TLRs described in humans recognizes a different spectrum of pathogen-associated molecular conformations. After interaction with pathogens, TLRs induce the innate immune response. They are involved in balancing Th1 versus Th2 immune responses, shifting the balance towards a Th1 response [9] TLRs are expressed in immune cells such as antigen-presenting cells, and in epithelial cells [24]. Polymorphisms in *TLRs* have been linked to childhood asthma [25-28].

In this systematic review we summarize research on CD14 and TLRs in relation to asthma in Caucasian children. By selecting this specific group we expect more clarity in study outcome as conclusions can differ between ethnicities [29]. Consequently, a better insight into the relation between CD14 and TLRs and pathology of childhood asthma in Caucasians would be achieved. Further, we analyzed the effect of environmental factors on the relation between CD14 and TLRs and asthma in Caucasian children.

## Methods

### Search strategy

**Figure 1:**
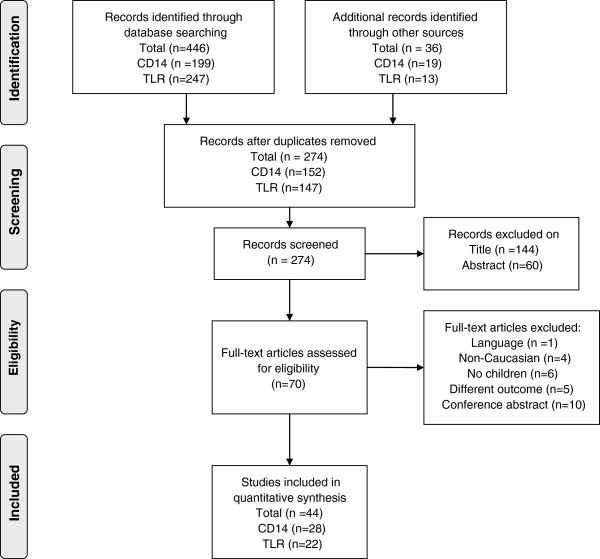
**PRISMA flow diagram of search.** The flow diagram depicts the flow of information through the different phases of the systematic review. It maps out the number of records identified, included and excluded, and the reasons for exclusions.

PubMed and EMBASE were searched for relevant articles up to October 2012 by two independent investigators. Primary search terms (CD14, toll-like, TLR) were combined with secondary search terms (asthma*, wheez*) and limitation ‘child’ to find as many relevant publications as possible. Articles were included if they concerned asthma in Caucasian children and investigated the relation with CD14 or TLRs. Articles were excluded if the study population was non-Caucasian (based on area where the study was conducted and stated by the authors) or if the full text was not available in English. Also, conference abstracts and reviews were excluded. The articles were screened by the first two authors for suitability first by title, later by abstract, and finally by full text. In case of disagreement, the senior author was asked to screen the article as well. Additionally, some studies were identified by a manual search of references of included studies. A PRISMA flow diagram of the search is included in Figure 1[30].

### Quality assessment

The first two authors independently assessed the quality of the selected studies. Quality assessment was carried out using the Newcastle-Ottawa quality assessment scale which has especially been developed for assessment of observational studies in systematic reviews [31]. In case criteria were met, a “*” was assigned. In case criteria were not met, a “-” was assigned. The overall quality of each article was assessed as better than average (+), average (+/−), or less than average (−). In case of disagreement the senior author was asked to score the article as well. We did not attempt to pool the data.

The categories for cohort studies were as follows: S1: Representativeness of the exposed cohort, S2: Selection of non-exposed cohort, S3: Ascertainment of exposure, S4: Outcome of interest not present at start of study, C1a/C1b: Comparability of cohorts on the basis of the design or analysis, O1: Assessment of outcome, O2: Duration of follow-up, O3: Adequacy of follow up. The categories of case control studies were as follows: S1: Case definition, S2: Representativeness of the cases, S3: Selection of controls, S4: Definition of controls, C1a/C1b Comparability of cases and controls on the basis of the design or analysis, E1: Ascertainment of exposure, E2: Same method of ascertainment for cases and controls, E3: Non-response rate. As no cross-sectional assessment tool was available in the Newcastle-Ottowa quality assessment scale, cross-sectional studies (as identified in the method section of the respective article) were assessed in both the cohort scale and the case control scale. Nested case control studies were scored as case control studies. Polymorphisms, gene expression and/or levels were analyzed by a standard method in all studies. Therefore ascertainment of exposure (S3 for cohorts, E1 and E2 for case control studies) was only scored in case of exposure to environmental factors. Also, we assigned a “*” for E3 in case control studies if the non-response rate was mentioned. Five articles could not be scored as they had neither cohort nor case control or cross-sectional design [19,32-34].

## Results

Of the 274 collected records, a total of 44 articles were included in this study (Figure 1). Only one article was excluded based on language [35]. Characteristics of included studies are provided in Additional file 1 (see Additional file 1). The results of the quality scoring can be viewed in Table 1 for cohort studies, and in Table 2 for case control studies.

**Table 1:** Quality scoring based on the Newcastle-Ottawa quality assessment scale: cohort studies

**First author**	**Year**	**S1**	**S2**	**S3**	**S4**	**C1a**	**C1b**	**O1**	**O2**	**O3**	**OQ**
Bieli † [10]	2007	*	*	*	-	*	*	-	*	-	+/−
Bottema [26]	2010	*	*	NA	*	-	-	-	*	-	+/−
Custovic [62]	2011	*	*	-	*	*	*	-	*	*	+
Eder † [25]	2004	*	*	*	-	*	*	-	*	-	+/−
Ege † [22]	2007	*	*	*	-	*	*	-	*	-	+/−
Fageras Bottcher † [39]	2004	-	*	*	-	*	*	*	*	-	+/−
Guerra [11]	2004	*	*	-	*	*	*	-	-	-	+/−
Jones [48]	2002	-	*	NA	*	-	-	-	-	-	–
Kabesch † [13]	2004	*	*	NA	*	-	-	-	*	-	+/−
Kerkhof [56]	2010	*	*	*	*	*	*	-	*	-	+
Lange [63]	2011	*	*	NA	-	*	*	*	*	-	+
Lodrup Carlsen [51]	2010	*	*	NA	*	*	*	-	*	*	+
O’Donnell [36]	2004	*	*	NA	-	*	*	-	*	*	+
Reijmerink [41]	2010	*	*	NA	*	-	-	-	*	-	+/−
Rothenbacher [53]	2005	*	*	*	*	*	*	-	-	*	+
Simpson [8]	2006	*	*	*	*	*	*	-	*	*	+
Snijders † [52]	2006	*	*	*	*	*	*	-	-	*	+
Soferman [54]	2004	-	*	NA	*	*	*	*	*	*	+

S1: Representativeness of the exposed cohort, S2: Selection of non-exposed cohort, S3: Ascertainment of exposure, S4: Outcome of interest not present at start of study, C1a/C1b: Comparability of cohorts on the basis of the design or analysis, O1: Assessment of outcome, O2: Duration of follow-up, O3: Adequacy of follow up (*: criteria were met; -: criteria were not met); OQ: Overall quality (+ better than average quality, +/− average quality, – less than average quality); NA = not applicable; † cross-sectional study.

**Table 2:** Quality scoring based on the Newcastle-Ottawa quality assessment scale: case control studies

**First author**	**Year**	**S1**	**S2**	**S3**	**S4**	**C1a**	**C1b**	**E1**	**E2**	**E3**	**OQ**
Bieli † [10]	2007	-	*	*	-	*	*	*	*	*	+
Bjornvold [38]	2009	*/-	*	*	*	-	-	NA	NA	-	+/−
Choudhry [40]	2005	-	-	*	-	*	*	-	*	-	+/−
Daley [21]	2009	-	*/-	*	-	-	-	NA	NA	-	+/−
Eder † [25]	2004	-	*	*	-	*	*	*	*	-	+/−
Ege † [22]	2007	-	*	*	-	*	*	*	*	*	+
Fageras Bottcher † [39]	2004	*	-	-	-	*	*	*	*	-	+/−
Genuneit [46]	2009	-	*	*	*/-	*	-	NA	NA	-	+/−
Heinzmann [12]	2003	*	*	*	-	-	-	NA	NA	-	+/−
Heinzmann [58]	2010	*	-	*	-	-	-	NA	NA	-	+/−
Hoffjan [61]	2005	-	-	-	*	-	-	NA	NA	-	–
Hussein [59]	2012	*	*	-	*	-	-	NA	NA	-	+/−
Jackola [23]	2006	*	-	*	*	*	-	NA	NA	-	+/−
Kabesch † [13]	2004	-	*	*	-	-	-	NA	NA	-	+/−
Kormann§ [27]	2008	*	*	*	*	*	-	NA	NA	-	+
Kurowski [44]	2011	-	-	*	-	-	-	*	*	-	–
Marcos [50]	2010	*	-	-	*	-	-	*	-	-	–
Miedema§ [45]	2012	-	-	*	*	-	-	NA	NA	-	+/−
Moller-Larsen [28]	2008	*	-	*	*	*	-	NA	NA	-	+/−
Perin [42]	2011	*	-	-	*	-	-	NA	NA	-	+/−
Puthothu [57]	2006	*	-	*	-	-	-	NA	NA	-	+/−
Schubert [60]	2006	*	-	*	-	-	-	NA	NA	-	+/−
Sengler § [37]	2003	-	*	*	*	*	-	NA	NA	-	+/−
Snijders † [52]	2006	-	*	*	*	*	*	*	*	-	+
Sorensen § [47]	2009	-	*	*	*	*	*	*	*	*	+
Tremblay [20]	2008	-	*/-	*	-	-	-	NA	NA	-	+/−
Yang [55]	2004	-	-	*	*	*	*	NA	NA	-	+/−
Zhang [43]	2011	*	*	*	*	*	*	*	*	-	+

S1: Case definition; S2: Representativeness of the cases; S3: Selection of controls; S4: Definition of controls; C1a/C1b Comparability of cases and controls on the basis of the design or analysis; E1: Ascertainment of exposure; E2: Same method of ascertainment for cases and controls; E3: Non-response rate (*: criteria were met; -: criteria were not met); OQ: Overall quality (+: better than average quality, +/− average quality, – less than average quality); † cross-sectional study; § nested case control study; NA = not applicable.

### Limited evidence for a relation between polymorphisms of *CD14* and childhood asthma

The relation between childhood asthma and polymorphisms in *CD14* was studied in 17 studies [10,12,13,19-21,34,36-45]. In most studies asthma was taken as outcome. In two studies asthma severity was taken as outcome [19,34]. Age ranged from 0 up to 19 years of age. The number of cases varied from 10 to 644, and controls from 115 to 1858 subjects. *CD14* rs2569190 polymorphism, also known as C-260T or C-159T, was studied in 16 studies [10,12,13,19-21,34,36-40,42-45]. Only one study found an association of this polymorphism with childhood asthma, showing an association of the T allele with decreased asthma severity (Table 3) [19]. For the rs4914 polymorphism an association was indicated for decreased asthma incidence in one of the two children cohorts (Study of Asthma Genetics and Environment Cohort) included in one study (Table 3) [20,21]. However, another study investigating rs4914 found no association [38]. All other polymorphisms studied did not show any association with asthma [10,12,20,21,38,40,41,43-45]. Partial duplication in studied population and studied single nucleotide polymorphisms was present between two studies [20,21].

**Table 3:** Single nucleotide polymorphisms with association with asthma

**Gene**	**rs-number**	**Alleles**	**Number of associations**	**Minor allel related to**	**Association ref.**	**No association ref.**
*CD14*	rs2569190	C > T	1 out of 15 (duplication [20,21])	decreased asthma severity	[19]	[10,12,13,20,21,34,36-40,42-45]
*CD14*	rs4914	G > C	1 out of 2 (duplication [20,21])	decreased asthma incidence	[20,21]	[38]
*TLR1*	rs5743595	T > C	1 out of 1	atopic asthma	[27]	NA
*TLR1*	rs4833095	A > G	1 out of 1	atopic asthma	[27]	NA
*TLR2*	rs4696480	A > T	3 out of 5 (partial duplication [26,56])	asthma/current asthma symptoms	[25,26,56]	[27,45]
*TLR2*	rs3804099	T > C	1 out of 6 (partial duplication [26,56])	nonatopic asthma (inverse)	[27]	[25,26,38,45,56]
*TLR2*	rs3804100	T > C	1 out of 6 (partial duplication [26,56])	allergic asthma (inverse)	[38]	[21,25,26,45,56]
*TLR2*	rs1898830	A > G	1 out of 4	asthma	[26]	[21,27,56]
*TLR2*	rs5743708	G > A	1 out of 2	asthma severity in atopic children	[59]	[43]
*TLR4*	rs2737190	A > G	1 out of 1	(non-atopic) asthma	[27]	NA
*TLR4*	rs4986790	A > G	3 out of 10	asthma, atopic asthma and asthma severity in atopic children	[34,39,59]	[41,43-45,55,56,60]
*TLR4*	rs4986791	C > T	1 out of 4	mild atopic asthma	[34]	[27,56,60]
*TLR6*	rs6531666	T > C	1 out of 1	asthma	[45]	NA
*TLR6*	rs5743789	T > A	1 out of 1	atopic asthma	[27]	NA
*TLR6*	rs5743798	C > T	1 out of 1	asthma	[45]	NA
*TLR6*	rs5743810	C > T	2 out of 5	(atopic) asthma	[27,61]	[21,45,57]
*TLR7*	rs179008	A > T	1 out of 2	asthma	[28]	[27]
*TLR8*	rs2407992	G > C	1 out of 1	asthma	[28]	NA
*TLR9*	rs187084	T > C	1 out of 3	asthma	[27]	[21,45]
*TLR10*	rs4129009	A > G	1 out of 2	atopic asthma inverse	[27]	[45]
*TLR10*	rs11096957	A > C	1 out of 3	asthma	[21]	[45,57]

Ref: reference, TLR: Toll-Like Receptor, NA: not applicable.

In three studies the association between rs2569190 in *CD14* and wheeze was addressed [8,46,47]. The age of the population studied varied from 18 months up to 13 years. One study investigated two additional polymorphisms (rs5744441 and rs5744455) [46]. None of these studies found a relationship between *CD14* polymorphisms and childhood wheeze.

### The relation between polymorphisms of *CD14* and childhood asthma can be influenced by environmental exposures

The protective effect of farm milk consumption was increased in carriers of one or, even more so, two major alleles of rs2915863 [10]. Also, in individuals exposed to tobacco smoke, a significant association was found between rs2569190 and rs3776138 polymorphisms and asthma severity [40]. Eastern (Russian) or Western (Finnish) environment/lifestyles did not influence the relationship of rs2569190 and rs5744455 on asthma ever in childhood [43]. Neither did age, breastfeeding, allergies to mold, house dust mites or pollens, and exposure to cats and dogs or other large animals in combination with rs5744454, rs2569190 and rs3776138 and asthma-related traits [40], nor dust or living area (rural or urban) with rs2569190 or rs3138078 and asthma [44]. Finally, a dose-dependent relation of endotoxin with non-atopic wheeze for individuals with homozygosity of the major allele of rs2569190 was shown [8]. The association between smoking or pet keeping and the risk of recurrent wheeze was not affected by the rs2569190 polymorphism [47].

### Polymorphisms in *CD14* lead to increased levels of sCD14

Several studies have shown that sCD14 levels are higher in the presence of one or two minor allele(s) of rs2569190 in chronic and also acute asthma in children [8,13,19,33]. In addition, during asthma attacks an inverse correlation was shown between asthma severity scores and the log value of sCD14 for the major allele of rs2569190 [19]. No significant association was found between rs574441, rs2569193, and rs2569190 and gene expression of CD14 [10].

### Higher levels of sCD14 are related to childhood asthma depending on disease status and time of measurement

Levels of sCD14 in cord blood were significantly higher in children with transient-atopy (positive skin prick test at two years of age but not at five years of age and without asthma), compared with never-atopic or persistent-atopic children. However, no changes between transient-atopy, never-atopic or persistant-atopic children could be shown in plasma sCD14 at six months, one year, and five years of age [48]. Also, levels of sCD14 were significantly higher during acute asthma attacks than at recovery in children with status asthmaticus [32]. However, these results might be biased by the administration of corticosteroids during the acute stage which might have caused suppression of cell activation, thereby reducing sCD14 levels in serum [49]. Multiple studies showed no association of sCD14 levels with asthma as measured in cord blood, serum or bronchoalveolar lavage fluid [23,50,51]. In one study no association of sCD14 levels in breast milk with wheeze was found [52].

### (Expression) levels of (s)CD14 can be related to environmental exposures leading to a different disease risk

One study showed that of the protective farm related factors that were associated with asthma (pig farming, farm milk consumption, frequent stay in animal sheds, and child’s involvement in haying), only being a farm child in general was associated with higher expression levels of CD14 [22]. Breastfed children who received breast milk with high sCD14 levels had less chance to develop asthma, reaching statistical significance in children of mothers with no history of atopic disease [53]. Another study showed that age or gender did not influence sCD14 production in asthmatic families [23].

CD14 serum levels in infants without recurrent wheezing was significantly higher than in wheezing infants, twelve months after hospitalization due to RSV-induced bronchiolitis. Also, in hospitalized patients sCD14 levels increased with age of admission [54].

Overall, results suggest that some environmental exposures like being a farm child or breast milk with high sCD14 levels protect against development of asthma, however, the number of studies is limited.

### Some evidence for a relation between polymorphisms of *TLRs* (2, 6, 9, 10) and childhood asthma

Childhood asthma in relation to polymorphisms in *TLR*s was addressed in 19 studies [21,25-28,34,38,39,41,43-45,55-61]. The age of the population in these studies ranged from 0 to 18 years with 31 to 644 cases and 184 to 2927 controls. Outcome was defined as atopic/allergic asthma, non-atopic asthma, bronchial asthma, current asthma symptoms or asthma in general.

*TLR4* is by far the most studied of all *TLR*s in relation to asthma in children, but only some studies showed an association (Table 3). Rs4986791 was statistically more frequent in mild atopic asthmatics [34], whereas rs2737190 showed an association with asthma in general and also non-atopic asthma [27] Rs4986790 showed no association with asthma in seven studies [41,43-45,55,56,60], but was associated with symptoms of (atopic) asthma in two other studies [34,39]. Also, rs4986790 was associated with increased asthma severity in atopic children [59]. However, in most studies polymorphisms of *TLR4* did not show any association with asthma [21,25,27,38,41,44,45,55],[56,59,60]. Some duplication between the studies of Reijmerink et al. and Kerkhof et al. was present as the same population was used for single nucleotide polymorphism analysis [41,56]. Studied polymorphisms in the study of Reijmerink were not specified [41].

*TLR2*, *TLR6*, *TLR9*, and *TLR10* have been associated with asthma in several studies. For *TLR2*, rs4696480, rs3804099, rs3804100, and rs1898830 showed association with childhood asthma in a number of studies, but not in others (Table 3) [21,25-27,38,43,56,62]. Moreover, rs5743708 was associated with increased asthma severity in atopic children (Table 3) [59]. Finally, rs13150331, rs4696483, rs5743704, rs7656411, rs1339, rs2289318, and rs5743708 showed no association with childhood asthma [21,43,59]. Two studies had an overlap in data [26,56]. For *TLR6*, rs6531666, rs5743789, rs5743798, and rs5743810 significant associations with asthma and atopic asthma were found, but not with asthma in other studies (Table 3) [21,27,45,57,61]. Haplotype spanning of polymorphisms in *TLR6* and *TLR10* (see *TLR10*) revealed an association with childhood asthma, indicating no individual but a combined influence of these polymorphisms on asthma in children [21,57]. Other *TLR6* polymorphisms were not associated with asthma in children [21,27,45]. For *TLR9*, rs187084 showed a positive association with childhood asthma (Table 3) [27], but not in all studies [21,45]. Other polymorphisms revealed no association with childhood asthma [21,27,44,45,60,63]. For *TLR10*, out of the eight studied polymorphisms, rs4129009 and rs11096957 showed association with (atopic) asthma in some studies (Table 3) [21,27,45,57]. The rs11096957 polymorphism only demonstrated association in one of the two children cohorts (Study of Asthma Genetics and Environment Cohort) studied by Daley et al [21]. Several polymorphisms (rs4274855, rs10856839, rs11096957, rs11096956, rs11096955, rs11466657, rs4219009) showed no individual association with bronchial asthma, however, haplotype spanning together with polymorphisms in TLR6 (rs5743794, and rs5743810) did [57]. Also rs4274855, rs10856839, rs11096957, and rs4129009 demonstrated a haplotype-based association [58].

*TLR7* and *TLR8* were only studied in two studies and *TLR1*, *TLR3*, and *TLR5* in one study: For *TLR7*, associations with asthma phenotypes for rs179008 separately, and rs179008, rs5743781, rs864058 haplotypes were observed in one study, however, rs179008 showed no association in another study (Table 3) [27,28]. For *TLR8*, association with asthma phenotype in childhood was shown for rs2407992 separately, and rs5741883, rs3764879, rs3764880, rs5744077, rs2159377, and rs2407992 haplotypes, but not rs3761624 (Table 3) [27,28]. For *TLR1*, rs5743595, rs4833095, and not rs5743594 showed significant inverse effects with atopic asthma in childhood (Table 3) [27]. For *TLR3*, rs3775291 showed no association with childhood asthma [27]. Also, rs5744168, rs2072493 and rs5744174 of *TLR5* showed no significant association with childhood asthma [27].

One study with 1105 cases and 3137 controls aged 8 to 12 years used wheeze in the past 12 months as outcome variable [46]. For *TLR4*, rs11536896 showed association, but three other polymorphisms did not. In *TLR2*, rs1898830 showed association with more frequent wheeze. For *TLR9*, out of the three studied polymorphisms, rs187084 showed positive association with wheeze.

### The relation between polymorphisms of *TLRs* and childhood asthma is dependent on environmental influences

Four studies addressed the relation between *TLR* polymorphisms and environmental exposure on childhood asthma [25,43,44,56]. The polymorphisms rs4696480 and rs1898830 in *TLR2* and the polymorphisms rs2770150, rs10759931, rs6478317, rs10759932, and rs1927911 in *TLR4* increased the effect of fine particulate matter exposure on the prevalence of doctor-diagnosed asthma from birth up to eight years of age. Polymorphism rs10759931 in *TLR4* showed an interaction between childhood asthma symptoms and air pollutant levels [56]. High or low endotoxin exposure revealed no interaction with rs4696480, rs3804099, and rs3804100 in *TLR2* for asthma diagnosis or current asthma symptoms in children [25]. There was no relation between *TLR2* rs5743708 or *TLR4* rs4986790 on asthma ever when Eastern or Western environment was taken into account [43]. A relationship neither existed for *TLR4* rs4986790 or *TLR9* rs352140 with asthma when dust or area (rural/urban) was taken into account [44].

Also, for polymorphism rs4696480 in *TLR2* a significant interaction was shown with day care attendance on the development of atopic wheeze in a longitudinal analysis from birth until the age of five years [62].

### Polymorphisms in *TLRs* relate to an increased gene expression of TLRs

Minor alleles of *TLR1* rs5743595, *TLR6* rs5743789, and *TLR10* rs4129009 polymorphisms were associated with increased mRNA expression of the respective *TLR*s in children [27]. In asthmatic children, farm related factors in combination with *TLR* polymorphisms were related to higher expression levels of respective TLRs in children [22].

## Discussion

We reviewed the literature on the relationship between CD14 and TLRs (polymorphisms) and asthma in Caucasian children. Although a number of studies suggest such a relation, the reviewed literature does not provide convincing evidence for a significant direct role of *CD14*. *TLR2*, *TLR6*, *TLR9*, and *TLR10* did show moderate association with asthma in childhood, but *TLR4* did not.

*CD14* rs2569190 may be exemplary for the relevance of polymorphisms in relation to asthma. This polymorphism has been analysed in two meta-analysis [64,65]. No significant overall association was detected between rs2569190 and asthma. However, when analysis was stratified by atopy, there was an association indicating a protective effect of the T-allele of rs2569190 in relation to atopic asthma [65]. This was also the case when the analysis was stratified by atopy and restricted to children [64]. However, these results should be interpreted with caution because of the exclusion of studies and the small sample sizes. Also, as the current review was restricted to Caucasian children, comparison is difficult. The lack of similarity in the set-up and populations of these studies makes deduction of conclusions difficult, even more so if the complexity of the disease is taken in account.

Although some TLRs showed associations with asthma in childhood, the most investigated TLR4 did not. A possible explanation is that TLR4, contrary to TLR2, TLR6, TLR9 and TLR10, mainly plays a role in immune response to gram-negative bacteria [25,61]. As the other TLRs are involved in the immune response to multiple microbial products, it can be expected that their role in heterogeneous symptoms and/or diseases is easier to establish than TLR4 [25,27,56,61].

Since CD14 and TLRs are the first mediators in the response of the innate immune system, it is plausible that their contribution to asthma development is influenced by environmental conditions. An increasing number of association studies have included such conditions. However, up to now, the evidence for an interplay between environment and polymorphisms of the mentioned genes is scanty and based on few observations only. If important environmental factors are not included, associations might be overlooked or misinterpreted. For example, it has been demonstrated that the direction of the relation between a CD14 polymorphism and asthma was dependent on endotoxin exposure [8]. Also interesting is the fact that sCD14 in serum seems to be increased at specific time points, which might indicate a time window at which children are more vulnerable for exposure [54]. Consequently, increasing the number of populations studied, without inclusion of environmental influences, will not guarantee more valid results. Indeed, one may argue that studies not taking environmental influences into account underestimate the complexity of the disease.

Another important development in recent years is the focus on haplotype analysis instead of individual polymorphisms [66-68]. It has been shown that the combined analysis of several polymorphisms by haplotypes increases the power to detect associations. In this review, two studies clearly show the strength of this approach, since in these studies individual polymorphisms did not provide an association, whereas haplotype spanning did [57,58]. Consequently, our insight into the role of CD14 and TLRs in relation to asthma would benefit from haplotype analysis. Application of this method will not demand a major investment since previous collected study populations can be reused.

Our review has several strengths. Firstly, by systematic evaluation of the available literature, our review provides an efficient and comprehensive summary of *CD14* and *TLR*s polymorphisms in relation to childhood asthma. Secondly, to increase the number of articles included, we choose to include studies with various outcome measures of asthma. Thirdly, independent searching and scoring of articles was performed to come to a balanced opinion. Also, no articles were rejected based on assigned score, as there is no general agreement on evaluation of observational studies [69]. Fourthly, we decided to focus on asthma as outcome measure as it is a more established entity than for example wheeze. While some studies only used parent reported diagnosis, other studies used more objective methods like doctor diagnosis and lung function [10,12,23,36,38,56]. However, as wheeze is a good predictor in older age for presence of asthma we included studies with outcome wheeze in our review as well. Though, we did separate the used definitions as the underlying mechanisms can differ. Finally, contrary to most reviews, the population included is restricted to Caucasian children.

Some limitations need to be mentioned. Firstly, the number of articles on this subject is limited. Secondly, the wide scatter in age within and between studies may lead to difficulties in interpretation of results due to such factors as behaviour and environment. Thirdly, as is the case for most reviews, the comparability between the studies was diminished by methodological differences between the studies.

## Conclusions

All studies taken together, no clear role for CD14 and TLRs in childhood asthma can be determined based on this review. This is probably due to unknown underlying confounding or mediating environmental factors. Another reason can be the fact that analysis has mostly been performed on individual polymorphisms instead of haplotype spanning. Therefore, planning future studies should include haplotype analysis and take environmental factors into account. Also, to be able to identify and confirm associations and gene-gene and gene-environmental interactions with sufficient power, large populations will be necessary. If the above mentioned suggestions are taken into consideration, future studies on the role of CD14 and TLRs in childhood asthma may be more successful.

## Abbreviations

sCD14: Soluble CD14; TLR: Toll-like receptor.

## Competing interests

The authors declare that they have no competing interest.

## Authors’ contributions

EK and BT contributed to the data-acquisition and writing of the manuscript, GE and ED were mainly involved in revision of the manuscript. QJ supervised the project. All authors substantially contributed to the design of the review, interpretation of data and revision of article. All authors read and approved the final manuscript.

## Supplementary Material

Additional file 1 **Characteristics of included studies.** Characteristics of included studies are provided: First author, year of publication, type of study, parameters studied, genetic variants included, outcome, environmental exposures, sample size, age of population, study setting, analysis technique, statistics used consistency with the Hardy-Weinberg equilibrium and genotyping success rates. Only data relevant for the current review are provided. Abbreviations in alphabetical order: ALEX: Allergy and Endotoxin, ANOVA: analysis of variance, CAMP: Childhood Asthma Management Program, CAPPS: Canadian Asthma Primary Prevention Study, Cc: case control design, Cs: cross-sectional design, Co: cohort design, DNBC: The Danish National Birth Cohort, ECA: Environment and Childhood Asthma, ELISA: Enzyme-Linked ImmunoSorbent Assay, FACS: Fluorescence-Activated Cell Sorting, FBAT: Family-Based Association Test, GEE: Generalized estimating equations, HWE: Hardy Weinberg Equilibrium, HPLC: High-Performance Liquid Chromatography, irt: in relation to, ISAAC: International Study of Asthma and Allergies in Childhood, ISS: Infant Immune Study, KOALA: Kind, Ouders en gezondheid: Aandacht voor Leefstijl en Aanleg, prospective birth cohort study in the Netherlands, LR: logistic regression, MAAS: Manchester Asthma and Allergy Study, MAS:Multicentre Allergy Study, MWU: Mann–Whitney U, NA: not applicable, NS: not stated, PARSIFAL: Prevention of Allergy - Risk Factors for Sensitisation in Children Related to Farming and Anthroposophic Lifestyle, PCR: Polymerase Chain Reaction, PIAMA: Prevention and Incidence of Asthma and Mite Allergy, PREVASC: Prevention of asthma in genetically susceptible children, RFLP: restriction fragment length polymorphism, RSV: Respiratory Syncytial Virus, SAGE: Study of Asthma Genes and Environment, sCD14: soluble CD14, SNP: Single Nucleotide Polymorphism, SPT: skin prick test, SSCP: single strand conformation polymorphism, TDT: transmission disequilibrium test, TLR: Toll-like receptor, USA: United States of America vs: versus, yrs: years, *X*^2^: Chi-square. Click here for file
